# Comparative Genomics across Three Ensifer Species Using a New Complete Genome Sequence of the Medicago Symbiont *Sinorhizobium* (*Ensifer*) *meliloti* WSM1022

**DOI:** 10.3390/microorganisms9122428

**Published:** 2021-11-25

**Authors:** Laura Baxter, Proyash Roy, Emma Picot, Jess Watts, Alex Jones, Helen Wilkinson, Patrick Schäfer, Miriam Gifford, Beatriz Lagunas

**Affiliations:** 1 School of Life Sciences, University of Warwick, Coventry CV4 7AL, UK; P.Roy@warwick.ac.uk (P.R.); E.Picot@warwick.ac.uk (E.P.); J.L.Watts@warwick.ac.uk (J.W.); alex.jones@warwick.ac.uk (A.J.); Helen.Wilkinson@warwick.ac.uk (H.W.); P.Schafer@warwick.ac.uk (P.S.); M.L.Gifford@warwick.ac.uk (M.G.); 2 Bioinformatics Research Technology Platform, University of Warwick, Coventry CV4 7AL, UK; 3 Department of Genetic Engineering and Biotechnology, University of Dhaka, Dhaka 1000, Bangladesh

**Keywords:** rhizobium, *Sinorhizobium*, *Ensifer*, rhizobial genome, *Medicago truncatula*, WSM1022, WSM419, Sm1021, nitrogen fixation, nodulation, nodule

## Abstract

Here, we report an improved and complete genome sequence of *Sinorhizobium* (*Ensifer*) *meliloti* strain WSM1022, a microsymbiont of *Medicago species*, revealing its tripartite structure. This improved genome sequence was generated combining Illumina and Oxford nanopore sequencing technologies to better understand the symbiotic properties of the bacterium. The 6.75 Mb WSM1022 genome consists of three scaffolds, corresponding to a chromosome (3.70 Mb) and the pSymA (1.38 Mb) and pSymB (1.66 Mb) megaplasmids. The assembly has an average GC content of 62.2% and a mean coverage of 77X. Genome annotation of WSM1022 predicted 6058 protein coding sequences (CDSs), 202 pseudogenes, 9 rRNAs (3 each of 5S, 16S, and 23S), 55 tRNAs, and 4 ncRNAs. We compared the genome of WSM1022 to two other rhizobial strains, closely related *Sinorhizobium* (*Ensifer*) *meliloti* Sm1021 and *Sinorhizobium* (*Ensifer*) *medicae* WSM419. Both WSM1022 and WSM419 species are high-efficiency rhizobial strains when in symbiosis with *Medicago truncatula*, whereas Sm1021 is ineffective. Our findings report significant genomic differences across the three strains with some similarities between the meliloti strains and some others between the high efficiency strains WSM1022 and WSM419. The addition of this high-quality rhizobial genome sequence in conjunction with comparative analyses will help to unravel the features that make a rhizobial symbiont highly efficient for nitrogen fixation.

## 1. Introduction

The legume–rhizobia symbiosis is one of the most studied plant–microbe interactions to date. Both the legume and the rhizobia can grow independently, but when they interact, the rhizobia converts atmospheric dinitrogen (N_2_) to a biologically usable form for the plant (e.g., NH_4_^+^), and in exchange, it receives carbon compounds. This process—if thoroughly understood—can contribute enormously to the sustainable agriculture goals [1,2]. Earlier studies in this area were centred on *Medicago sativa* (alfalfa or lucerne), a widely cultivated forage crop, and one of its efficient microsymbionts, *Sinorhizobium meliloti* 1021 (Sm1021), that lead to the complete genome sequencing of Sm1021 in 2001 [3]. On the plant side, however, the focus shifted from *M. sativa* to *Medicago truncatula* as the model legume due to (amongst other reasons) its simple diploid genetics, comparatively smaller genome, and shorter generation time [4,5,6]. *M. truncatula* was known to form nodules (specialised root organ where rhizobia colonise) with Sm1021 [5], a spontaneous streptomycin-resistant derivative of Sm2011 [7], but later studies reported the interaction as only partially effective [8,9]. Sm1021 was shown to be a poor match for nitrogen fixation with numerous *Medicago* spp., including *M. truncatula*. Sm1021 was, therefore, deemed as an inefficient model for studying legume–rhizobia symbiosis, especially after the development of *M. truncatula* as the model legume. Despite this, Sm1021 is still widely used as a model strain in laboratories around the world due to its genetic tractability and the existence of an array of mutants.

Another *S. meliloti* strain, known as WSM1022 (originally isolated from *M. orbicularis* in 1987), was found to form highly efficient symbiosis (over 80% shoot growth compared to N-fed controls) with different *M. truncatula* accessions and some other *Medicago* spp. [8]. By contrast, Sm1021 was only suboptimal (less than 40% shoot growth) for those species [9]. WSM1022 effectiveness with *M. truncatula* made it a better inoculant in legume–rhizobia research, prompting the sequencing of its whole genome in 2013 [10]. The resulting draft assembly comprised 125 contigs arranged into 121 scaffolds, with 6323 predicted coding sequences.

Another species within the *Sinorhizobium* genus, *S. medicae* WSM419, has a comparatively promiscuous approach towards different *Medicago* spp., including *M. truncatula* [8]. WSM419 was originally isolated in 1981 from the nodules of *M. murex*, an annual medic that both Sm1021 and WSM1022 fail to nodulate [11]. Terpolilli et al. found that WSM419 could efficiently fix nitrogen with *M. truncatula*. In common with WSM1022, WSM419 fixes about twice as much nitrogen as compared to Sm1021 when paired with *M. truncatula* A17 [8]. A well-sequenced genome for the WSM419 became available in 2010 [11].

Here, we present a complete genome of *S. meliloti* strain WSM1022, with a detailed description of its sequence and annotation. We also provide an initial comparative genome analysis to related strains Sm1021 and WSM419, providing insights into potential genetic determinants of efficient nitrogen fixation within the Sinorhizobium–Medicago symbiosis.

## 2. Materials and Methods

### 2.1. Strains Used in This Study

Strains *Sinorhizobium meliloti* Sm1021, *Sinorhizobium medicae* WSM419, and *Sinorhizobium meliloti* WSM1022 were kindly donated by Dr. Jason Terpolilli, Murdoch University, Perth, Australia. *Sinorhizobium meliloti* 1021 was originally obtained from Professor Sharon Long (Stanford University, Stanford, CA, USA). *Sinorhizobium medicae* WSM419 (Centre for Rhizobium Studies WSM Genebank) is an isolate from Sardinia, Italy. *Sinorhizobium meliloti* WSM1022 is a field isolate obtained from Naxos (Greece) from the annual legume *Medicago orbicularis* (J. G. Howieson, CRS, WSM Genebank).

### 2.2. Preparation of Biological Material for Sequencing

For DNA extraction, WSM1022 was grown on tryptone yeast (TY) agar for two days. Using a single colony, we inoculated and incubated TY broth medium cultures overnight on a rotary shaker (200 rpm) at 28 °C. For the standard Illumina sequencing performed, we followed the agar protocol established by MicrobesNG^®^ (Birmingham, UK). We mixed a single colony of the strain to be sequenced in 100 μL sterile PBS buffer and plated it on a TY agar plate. The plate was grown for two days at 28 °C. A picture of the plate was taken for visual quality controls. Using a large sterile loop, we took all bacterial culture off the plate and mixed it into the barcoded bead tube supplied by MicrobesNG^®^. The tube was mixed by inverting 10 times, sealed, and sent back to MicrobesNG^®^. For the enhanced nanopore sequencing, we followed the liquid broth protocol established by MicrobesNG^®^. We mixed a single colony of the strain to be sequenced in 200 μL sterile PBS buffer and plated 100 μL on a TY agar plate for visual quality controls. With the other 100 μL, a sterile broth of 25 mL was inoculated and incubated on a rotary shaker (200 rpm) at 28 °C until the upper exponential phase. Bacteria were then pelleted by centrifugation (10 min at 500× *g*). The cell pellet was weighed to ensure enough material had been collected (between 300 and 600 mg). Pelleted cells were resuspended in 500μL of the cryopreservant liquid from the barcoded bead tube supplied by MicrobesNG^®^ and transferred into the barcoded bead tube. This was sealed and sent back to MicrobesNG^®^. Library preparation and sequencing was performed by MicrobesNG^®^ using their enhanced genome service which combines Illumina short reads and Nanopore long reads. ONT basecalling was performed using guppy (ONT) version 3.0.6. Illumina sequencing was performed on a HiSeq sequencer, generating 1,300,674 paired-end short reads with an average length of 250 bp. Illumina adapters and low-quality sequences at the ends of reads were removed using trimmomatic version 0.39, leaving 12,731,115 paired sequences with a mean per-sequence quality score of 37.4. Oxford nanopore sequencing generated 24,155 reads with a mean length of 6.4 kb and a mean per-sequence quality score of 16.7.

### 2.3. Genome Assembly and Annotation

Reads were assembled de novo using a hybrid assembly approach with SPAdes (version 3.12.0) [12]. Reads were mapped back to the assembly using bwa (version 0.7.17-r1188, [13]) and mapping statistics generated with Qualimap [14]. Genome assembly metrics were calculated using QUAST [15]. Annotation was performed by means of the NCBI Prokaryotic Genome Annotation Pipeline (PGAP, [16]) upon submission to GenBank. Assembly and annotation completeness was assessed with BUSCO (Benchmarking Universal Single-Copy Orthologs, v5 [17]). Circular maps were generated using CGView [18]. Nucleotide skew was calculated and plotted using GenSkew (https://genskew.csb.univie.ac.at/ (accessed on 10 October 2021)), part of MIPS [19]. ISEScan (version 1.7.2.1) was used to predict IS elements [20]. Prophage sequences were identified using PHASTER [21]. EffectiveDB was used to predict secreted proteins and secretion systems [22]. Orthologous cluster analysis was performed with OrthoVenn2 [23].

### 2.4. Data Availability

The whole genome sequence of *Sinorhizobium meliloti* strain WSM1022 was deposited at NCBI under BioProject PRJNA636618 with BioSample SAMN02597176, and GenBank/RefSeq assembly accession GCF_0013315775.1.

### 2.5. Comparative Genomics

Average nucleotide identity was calculated with PyANI [24]. Multiple genome alignments were created with MAUVE.

## 3. Results

### 3.1. High-Quality Sinorhizobium Meliloti WSM1022 Genome Assembly and Annotation

**Figure 1:**
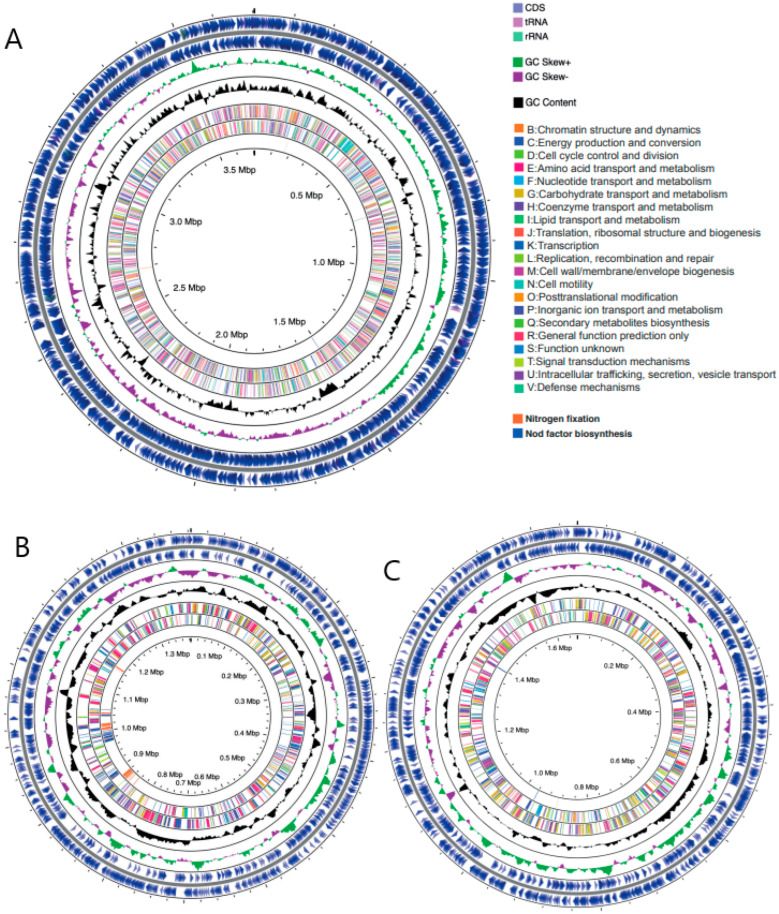
*Sinorhizobium meliloti* WSM1022 genome composition. Graphical circular maps of chromosome (**A**), pA (**B**) and pB (**C**), generated using CGView. From outside to centre, rings show CDS (lilac), tRNA (pink) and rRNA (sage green) on the forward and reverse strand; positive and negative GC skew (green and purple, respectively); GC content (black); COG category (colours in key) on forward and reverse strands; Nitrogen fixation genes (orange) and Nod factor biosynthesis genes; (blue); genome position in Mbp.

The final chromosome-level assembly consists of 6,751,834 bases (Figure 1), and is tripartite in structure, comprising three scaffolds representing the chromosome (3.7 Mb, Figure 1A) and two megaplasmid sequences pA (1.38 Mb, Figure 1B) and pB (1.66 Mb, Figure 1C), with an average GC content of 62.22%. Illumina data provided an average 77.7X coverage, and Nanopore an average of 22.7X coverage of the genome. Our assembly is to the chromosome level, thus representing a significant improvement over the previous assembly consisting of 121 scaffolds. Assembly metrics of the two genome assembly versions are shown in Table S1 for comparison. Table S1 shows a comparison between the genome assemblies of the previous WSM1022 genome deposited and that done in this study. The higher genome quality was observed by the lower number of contigs and the higher value of N50, reflecting the complete contiguity of the new assembly. A total of 110,268 previously unsequenced bases in the genome were determined, gaps were closed, and ambiguous/N bases were resolved.

The NCBI Prokaryotic Genome Annotation Pipeline (PGAP) predicted 6058 protein coding sequences (CDSs) with an average length of 313aa, 202 pseudogenes, 9 rRNAs (3 each of 5S, 16S, and 23S), 55 tRNAs, and 4 ncRNAs (Table 1). Low-quality sequences leading to poor assembly can lead to an inflated gene number and increase in pseudogenes, which was not observed in this study [25,26]. Additionally, BUSCO (Benchmarking Universal Single-Copy Orthologs) reported 100% assembly and annotation completeness in both genome and protein mode when benchmarking against the rhizobiales lineage dataset (637 complete single-copy BUSCOs and 2 complete and duplicated BUSCOs). This indicates orthologs of all highly conserved single-copy genes expected in rhizobiales were present in our assembly. Using information from the database of Clusters of Ortholgous Groups of proteins (COG), we assigned putative functional terms to 89.6% of CDS (Figure 1), with the remaining sequences consisting of homologs to hypothetical proteins or contained domains of unknown function. WSM1022 contains a full complement of nod factor biosynthesis and nitrogen fixation genes (Figure 1).

GC content was unevenly distributed across the genome at several locations, and GC skew ((G − C)/(G + C) showed a marked asymmetrical distribution in each of the replicons (Figure 1 (green/purple shown in ring 2) and Figure S1), typical of bacterial genomes. This segregates the replicons into two regions: one with an excess of G over C corresponding to the leading strand, and the other with an excess of C over G corresponding to the lagging strand. The transition points of the GC skew graph provide putative locations for the origin of replication (minimum) and the terminus (maximum). Supplementary calculations were performed with GenSkew, giving these locations as base positions 3,403,057 and 1,444,873 in the chromosome, 67,767 and 912,780 in pA, and position 464,256 and 1,088,256 in pB (Figure S1). In contrast to the chromosome and pB, the GC content and GC skew of pA exhibited a noisier distribution, possibly correlating with the presence of mobile elements within that replicon.

### 3.2. Genomic Comparisons across the Three Rhizobial Strains

#### 3.2.1. General Genome Feature Comparisons across the Rhizobial Strains in This Study

Table 1 compares general genomic features of the three genomes. The genome of WSM1022 is 6.75 Mb, with a 3.7 Mb chromosome and two megaplasmids of 1.38 Mb (pA) and 1.66 Mb (pB). Sm1021 is very similar, with a 6.69 Mb genome comprising a 3.65 Mb chromosome, as well as two megaplasmids of 1.35-Mb (pSymA) and 1.68-Mb (pSymB). The 6.82 Mb WSM419 genome contains a 3.78 Mb chromosome, two megaplasmids of 1.57 Mb (pSMED01) and 1.25 Mb (pSMED02), and a third smaller plasmid (pSMED03, 0.22 Mb) not found in the other strains. The percentage of GC in the genome found for these species is in line with what was published before, with *S. medicae* species containing lower GC contents than *S. meliloti* [27].

To compare genetic relatedness among the three species, we calculated pairwise average nucleotide identity (ANI) between the genomes, as depicted in Figure S2A. Sm1021 and WSM1022 share 98.8% identity with each other but share less identity—88.2% and 88.3% in turn—with WSM419, clearly delineating the species-level distinction between the strains.

#### 3.2.2. Differences in Genome Feature Arrangement

**Figure 2:**
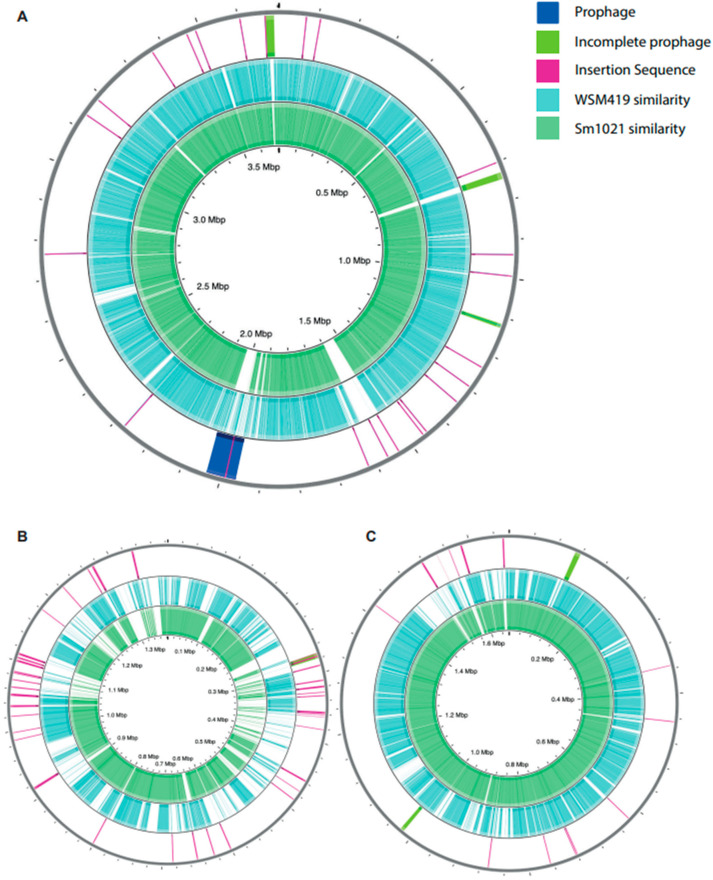
Comparative genomics of Sm1021, WSM1022 and WSM419. (**A**–**C**) Circular plots representing sequences of WSM1022 chromosome (**A**), pA (**B**) and pB (**C**). Innermost rings (green) show sequence similarity to corresponding replicon of Sm1021. Middle rings (light blue) show sequence similarity to corresponding replicons of WSM419. Outer rings show locations of IS and prophage sequences.

In order to broadly identify regions of similarity and difference at the genomic scale, we used BLAST to compare the corresponding chromosome or plasmid nucleotide sequences of each isolate within CGView, using WSM1022 as the reference strain (Figure 2A–C). Visual inspection of the alignment shows that the chromosomes of all three strains are highly conserved, displaying high levels of sequence similarity across the majority of the WSM1022 sequence (Figure 2A). However, there are some regions of WSM1022 without significant sequence similarity to the other strains, notably 728–745 kbp, 1530–1595 kbp, and 1976–2032 kbp. The larger megaplasmids (denoted pB, pSymB, and pSMED01 in WSM1022, Sm1021, and WSM419, respectively) also show a high degree of sequence similarity (Figure 2C), especially between WSM1022 and Sm1021. The smaller megaplasmid sequences (denoted pA, pSymA, and pSMED02 in WSM1022, Sm1021, and WSM419, respectively) share fewer regions of similarity, with several regions being absent with respect to WSM1022 (Figure 2B). To explore the genomic landscapes in more detail, we made multiple sequence alignments with MAUVE. This confirmed that the chromosomes are composed of large, syntenic, co-linear blocks (Figure S2B). It also enabled the identification of a structural rearrangement/inversion in WSM419 pSMED01 relative to pB and pSymB in the other strains at position 100–150 kbp (Figure S2D). The smaller megaplasmids (pA, pSymA, and pSMED02) show a high degree of sequence variability between strains, with shorter co-linear blocks interspersed with regions of low-similarity sequence (Figure S2C).

#### 3.2.3. Insertion Sequences and Bacteriophages

Insertion sequences (IS) are mobile genetic elements known to be fairly abundant in plasmids or chormosomal islands that contain genes needed for symbiosis [28]. We used ISEscan to predict the IS content of each genome for comparison. Overall, the amount of bp covered by IS sequences varies between genomes: WSM1022 has 87 predicted elements, covering 122,992 bases (1.82% genome); Sm1021 has 126 predicted elements, covering 166,567 bases (2.49% genome); and WSM419 has 135 predicted elements, covering 176,865 bases (2.59% genome). All three genomes contain a significant number of IS, but their type, abundance, and distribution vary between the genomes/replicons, creating unique IS profiles (Figure 2A–C). In WSM1022, the pA plasmid hosts more IS than pB, which is consistent with the pattern observed in Sm1021 and WSM419, with pSymA and pSMED02 containing more IS than pSymB or pSMED01. Plotting the IS locations within WSM1022 (Figure 2A–C) revealed that they are not strongly conserved between species, frequently located in/associated with regions of sequence variability, typical of mobile genetic elements.

#### 3.2.4. Nod Factor Biosynthesis and Nitrogen Fixation Genes

**Figure 3:**
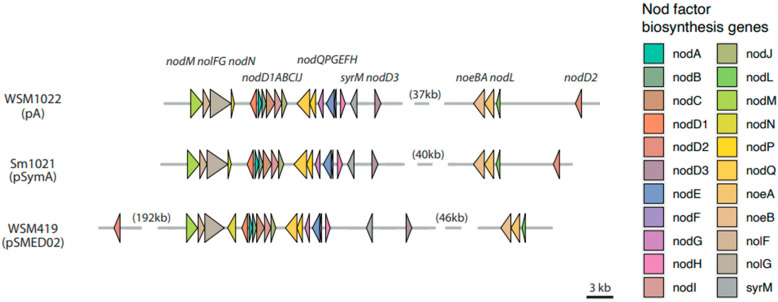
Conserved arrangement of Nod factor biosynthesis genes in plasmids of the three strains. Arrows indicate the genes encoding Nod factor biosynthesis genes present. Numbers in parentheses indicate distances between clusters of genes.

Nod factor biosynthesis genes (*nod*, *noe*, and *nol*) are involved in the synthesis of host-specific lipo-chito-oligosaccharide (LCOs) which are essential for initiation of symbiosis [29]. All three strains contain a full complement of most common nod genes [30], and by analysing the full genome assemblies of these strains, we can locate these genes into their correct order in the genome, and they exhibit a strikingly syntenic organization (the same genes in the same order). Figure 3 shows the physical arrangement of the Nod factor biosynthesis genes in the three species, located on pA, pSymA, and pSMED02 megaplasmids. Gene order is completely conserved (*nodM*, *nolFG*, *nodN*, *nodD1ABCIJ*, *nodQPGEFH*, *syrM*, *nodD3*, *noeBA*, *nodL*, *nodD2*), with the exception of nodD2 in WSM419, which has undergone rearrangement to a more distant location (192 kb upstream) with respect to the other genes in the cluster. In addition to these nod gene clusters, each strain has a small number of nod genes located on other partitions. Megaplasmids pB, pSymB, and pSMED01 contain an additional nodP_2_Q_2_ gene pair. The chromosomes of each strain encode an additional nodP and nodM gene. As expected, all three strains contain orthologs of the major nitrogen fixation genes (*nifH*, *nifD*, *nifK*, *nifA*, *nifBEN*, *fixABCX*, *fixNOPQ*, *fixLJ*, *fixK*, *fixGHIS,* and *fdxN*), predominantly co-located on megaplasmids pA, pSymA, and pSMED02 with the nod factor biosynthesis gene clusters [31].

### 3.3. Orthologous Protein Predictions

Another feature known to contribute to variation between species is the presence of bacteriophage genes. We examined the prophage content of each species using PHASTER. All replicons of all strains have at least one putative prophage region, but they differ in predictive score (intact, incomplete, or questionable), type, and number (Figure 2A–C). WSM419 has the greatest total predicted prophage content (215 kb), Sm1021 has the least (110 kb), and WSM1022 has 143 kb. WSM1022 contains an intact prophage region within its chromosome, containing tail, transposase, head, capsid, terminase, and integrase proteins. It has the strongest similarity to PHAGE_Sinorh_phiLM21_NC_029046, and this prophage region is also present in the chromosome of WSM419. By contrast, Sm1021 does not contain any intact (complete) prophage regions. The 1022 chromosome also has three incomplete prophages, which are not retained in the other genomes. The prophage profile on the WSM1022 plasmids, however, appear to be similar to those found on the corresponding plasmids of Sm1021 and WSM419.

**Figure 4:**
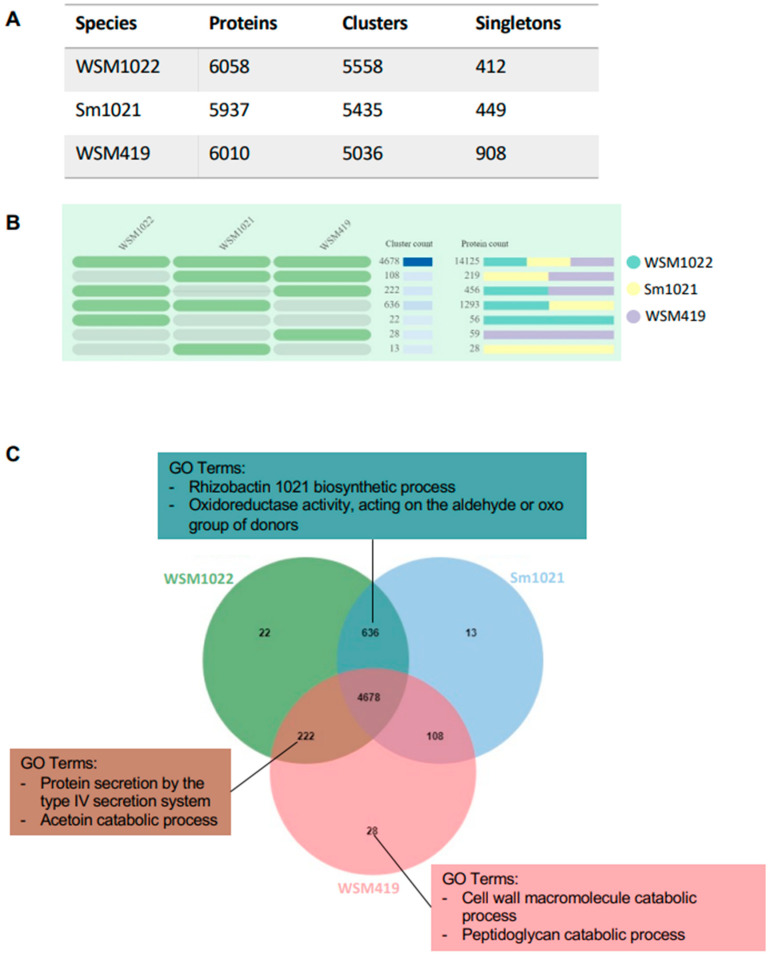
Orthovenn results on orthologous protein clusters in WSM1022, Sm1021 and WSM419. (**A**) Table indicating total number of proteins, number of clusters (highly similar protein sequences) and singletons (cannot be clustered) in each rhizobial species used in this study. (**B**) Presence of cluster groups in each rhizobial species, (dark green—presence, light green—absence). Cluster count represents the number of protein clusters within each cluster group (darker blue for higher protein cluster counts). Protein counts reflect the number of proteins within each protein cluster, colours refer to the distribution of those protein counts in each species. (**C**) Venn diagram showing the number of protein clusters identified in each species). Interesting GO Terms that were found enriched in certain cluster groups are also added and affixed to the associated region.

We next set out to investigate the common protein coding sequences between the strains. OrthoVenn2 is a web platform that uses a graph-based method for similarity comparison and annotation of orthologous gene clusters among multiple species [23]. OrthoVenn2 calculates pairwise sequence similarities between all input protein sequences defining orthologous clusters. These clusters are composed of highly similar protein sequences that, most likely, perform the same function. Those proteins that cannot be clustered with others from the same (or other) species in the analysis are classified as singletons. In our OrthoVenn2 analysis (summarised in Figure 4), we found a large group of 4678 common protein clusters to all three rhizobial species that most likely belong to core Sinorhizobium bacterial metabolism (Figure 4B,C). In this study, we found a greater number of core Sinorhizobium protein clusters than previously described [27], as we have focused in species that are highly related phylogenetically but that differ in their nitrogen fixation efficiency with Medicago species. When comparing Sm1021 and WSM1022, we found more common protein clusters than in any other comparison (636 in Sm1021/WSM1022, 108 in WSM419/Sm1021, and 222 in WSM419/WSM1022). The presence of higher cluster numbers in this comparison is probably in line with the fact that these two are meliloti species, whilst WSM419 is a medicae species.

#### 3.3.1. *Sinorhizobium meliloti* Orthologous Protein Clusters

When looking at the GO terms enriched in those 636 protein clusters, we found the biological process ‘rhizobactin 1021 biosynthetic process (GO:0019289)’ with six protein clusters (*p*-value 4 × 10^−7^) and the molecular function ‘oxidoreductase activity, acting on the aldehyde or oxo group of donors (GO:0016903)’ with five protein clusters (*p*-value 2.1 × 10^−4^; Figure 4, Table S2). Rhizobactin 1021 is a siderophore produced by *Sinorhizobium meliloti* that helps in capturing iron in free-living conditions, contributing to rhizosphere colonisation, swarming motility, and biofilm formation [32,33]. We cannot find genes associated with these GO terms in the WSM419 genome (Figure 4, Table S2); however, we cannot rule out that this strain could be producing a similar siderophore compound with a different protein set.

#### 3.3.2. Protein Clusters Putatively Linked to *Medicago truncatula* High-Efficiency Compatible Nitrogen Fixer Symbionts

The 222 common orthologous protein clusters between WSM419 and WSM1022 could potentially represent functions that are enriched in high efficiency strains or genomic features of strains that are highly compatible with *Medicago truncatula*. Interestingly, amongst these, we find protein secretion terms, including the GO term ‘protein secretion by the type IV secretion system (GO:0030255)’ which is enriched with eight clusters (*p*-value 6.8 × 10^−12^; Figure 4, Table S2).

These secretion systems have been known to fine-tune the interaction of bacteria with plant hosts, both in pathogenic and beneficial interactions [34]. Type III secretion systems are derived from flagella, and type IV are conjugation apparatuses, and they both conduct effectors from the bacterial to the plant cytoplasm. These secretion systems have been identified in some, but not all, rhizobial species [35] and have been described to help rhizobia evade plant immunity [36] and confer the ability to develop nodules in legumes [37]. We used tools within the EffectiveDB software suite to determine which components of the secretory systems are present in the genomes analysed. A major difference was found in the secretion systems present, with WSM1022 containing sufficient components to operate both Type III and Type IV secretion systems, whereas Sm1021 and WSM419 are only predicted to encode a functioning Type IV secretion system (Table 2). All the Type III components are located within a 16 kb region of the pA megaplasmid of WSM1022. By contrast, Sm1021 and WSM419 contain only FliI, which encodes for a flagellum-specific ATP synthase, and are chromosomally located. The Type IV components of WSM1022 are also located on pA but separate to the Type III components. The TypeIV components of Sm1021 are located solely on pSymA, whereas in WSM419, they are split between plasmids pSMED02 and pSMED03, reflecting a higher degree of recombination in the WSM419 genome relative to the other strains. The OrthoVenn results highlight the orthologous (based on sequence similarity) protein clusters, whilst the EffectiveDB tool performs a general analysis of the whole genome independently of the sequence similarity. Hence, these results point to a high degree of similarity between the secretion system related proteins in WSM1022 and WSM419, despite them belonging to different Sinorhizobium species. EffectiveDB is also able to predict the secretome of these strains. We found that all three strains contain a similarly large number of putative secreted proteins, with 1025, 1001, and 1063 in WSM1022, Sm1021, and WSM419, respectively.

Moreover, the other GO term enriched in this group of 222 protein clusters is ‘acetoin catabolic process’ (GO:0045150, *p*-value 0.011). Acetoin is a volatile rhizobacterial compound that elicits plant defence responses [38] and it has been described to be produced in biofilms and might contribute to prevent pathogen proliferation.

#### 3.3.3. Relevant Unique Protein Clusters and Singletons

Being the only medicae species in the analysis and containing an extra plasmid when compared to Sm1021 and WSM1022 (Table 1), WSM419 presents the largest number of singletons (908, Figure 4A) and the largest number of individual clusters (28, Figure 4B,C), and these might as well be medicae species-related proteins. When performing a GO term enrichment of the individual clusters and singletons for each rhizobial species, we observed some are still common terms for the three species (Table S2). For example, the GO terms ‘DNA integration’ (GO:0015074) and ‘transposition, DNA-mediated’ (GO: 0006313) are enriched in all three species individually, probably indicating that despite these terms being in individual clusters or singletons, the proteins in them most likely perform the same functions but do not meet the strict OrthoVenn requirements to belong in a cluster together.

When analysing those specific to WSM419, we found enrichment on the GO terms ‘cell wall macromolecule catabolic process’ (GO:0016998), ‘peptidoglycan catabolic process’ (GO:0009253), and lysozyme activity (GO:0003796). All these indicate a putative ability of WSM419 to degrade plant cell walls such as it has been proposed for other rhizobial species and that could be advantageous in the early stages of plant colonisation [39].

## 4. Discussion

Assembling the complete genome of WSM1022 reveals a genome with a tripartite structure, comprising a chromosome and two megaplasmids. This is consistent with the genome architecture of related strain Sm1021, and similar to that of WSM419, which contains an additional third plasmid. The chromosomes of the three strains exhibit high levels of sequence similarity and display strong synteny, barring some insertions. The megaplasmids (particularly pA/pSymA/pSMED02) display much greater sequence variability between strains, and as such are likely to be significant contributors to differences in environmental adaptability between the three strains. Interestingly the Nod factor biosynthetic genes exhibit a high syntenic organization in all three strains, with the exception of nodD2, which has suffered from more severe rearrangement in WSM419, probably reflecting the bigger phylogenetic distance of WSM419 (*S. medicae*) to WSM1022 and Sm1021 (both *S. meliloti*).

Using the OrthoVenn software, we analysed the orthologous proteins in these rhizobial species. The relationship between legume plants and rhizobia is dependent on iron, since this element is required for the nitrogenase complex, the synthesis of lehaemoglobins, and nodule development overall. The rhizobactin 1021 siderophore compound helps in capturing iron in free-living conditions, contributing to rhizosphere colonisation, swarming motility, and biofilm formation [32,33]. Other bacterial species produce similar compounds [40,41], including also some other rhizobial species such as Rhizobium leguminosarum [42], and the biosynthesis of all these compounds seems to be dependent on low iron availability [43]. According to our results, the rhizobactin biosynthesis pathway does not seem to be conserved in the *Sinorhizobium medicae* WSM419 species. Interestingly, when exposed to low iron conditions, WSM419 showed decreased growth rates in contrast to other rhizobium species such as rhizobium NGR234, *Rhizobium meliloti* U45, or *Rhizobium leguminosarum* WU235. This observation was independent of the fact that all species seemed to secrete siderophore compounds [44]. Therefore, despite the fact that we cannot rule out that WSM419 could be producing a similar siderophore compound with a slightly different protein set, it is possible that the lack of the ‘rhizobactin 1021 biosynthesis pathway’ in this strain accounts for its poor fitness in low iron conditions.

Results from the OrthoVenn analysis also indicate similarity between the secretion system-related proteins found in WSM1022 and WSM419, despite them belonging to different Sinorhizobium species. This result is interesting since the prediction of secretory pathway-related proteins using EffectiveDB shows no differences across strains in number of proteins related to this pathway. This analysis does show that WSM1022 is the only strain where both Type III and Type IV secretion systems coexist in the genome, whilst WSM419 and Sm1021 only contain Type IV. Type IV secretion systems can contribute to the transfer of large nucleic acids and proteins through the cell envelope, and they share similarities to the Agrobacterium Vir subunits [45,46]. Type III secretion systems are mostly studied in pathogenic bacteria; however, they have been studied in rhizobial partners [35,47] and are exploited by bacteria to supress or evade the host defence mechanisms. Homologues of the classical rhc genes in this secretion system have not been found on *S. meliloti* 2011 and are not believed to be ubiquitous [35]. The presence of an extra secretion system most likely provides this rhizobial strain with the ability to secrete a broader spectrum of molecules under different conditions or at different stages of nodulation, further fine-tuning the host’s response. Further analysis of the genomic features in beneficial rhizobia could elucidate how some strains establish some highly efficient symbiosis or perform better in certain field conditions (Lagunas et al., in preparation).

The complete genome of high efficiency strain WSM1022 and its detailed annotation provides a valuable resource for researchers, enabling the use of comparative genomics approaches to further unravel the genetic mechanisms underpinning the rhizobium–*Medicago truncatula* symbiotic interaction.

## Figures and Tables

**Table 1:** Comparison of general genome features of the rhizobial strains in this study.

	WSM1022	Sm1021	WSM419
Species	*S. meliloti*	*S. meliloti*	*S. medicae*
Genome size (bp, total)	6,751,834	6,691,694	6,817,576
No. of contigs	3	3	4
No. of chromosomes	1	1	1
No. of plasmids	2	2	3
GC content (%)	62.22	62.17	61.15
RefSeq/GenBank assembly accession	GCF_0013315775.1	GCF_00006965.1	GCF_000017145.1
Genes (total)	6328	6293	6464
CDS (total)	6260	6225	6396
CDS (with protein)	6058	5981	6068
Genes (RNA)	68	68	68
rRNAs (5S, 15S, 23S)	3, 3, 3	3, 3, 3	3, 3, 3
tRNAs	55	55	55
ncRNAs	4	4	4
Pseudo Genes (total, without protein)	202	244	328
Pseudo Genes (frameshifted)	134 of 202	173 of 244	218 of 328
Pseudo Genes (incomplete)	97 of 202	97 of 244	170 of 328
Pseudo Genes (internal stop)	18 of 202	28 of 244	41 of 326
Pseudo Genes (multiple problems)	45 of 202	49 of 244	94 of 326

**Table 2:** Type III and IV secretion system proteins present in each genome. EffectiveS346 predictions of presence/absence of Type III and Type IV secretion system components in the three genomes. Missing components are indicated by “-“. Chromosome/plasmid locations of the corresponding genes are indicated with background colours for plasmid pA/pSymA/pSMED02 (blue), chromosome (green) and pSMED03 (peach).

	COG ID	COG Symbol	WSM1022 Protein	Sm1021 Protein	WSM419 Protein
Type III	COG4669	EscJ	QKN17985.1	-	-
	COG4790	EscR/YscR	QKN17990.1	-	-
	COG4794	EscS/YscS	QKN17991.1	-	-
	COG4791	EscT/YscT	QKN17992.1	-	-
	COG4792	EscU/YscU	QKN17973.1	-	-
	COG4789	EscV	QKN17977.1	-	-
	COG1157	FliI	QKN17988.1	WP_010968730.1	WP_011974463.1
	COG1317	FliH	-	-	-
Type IV	COG3838	VirB2	QKN18893.1	WP_010967695.1	WP_011971086.1
	COG3702	VirB3	QKN18440.1	WP_010967694.1	WP_011971088.1
	COG3701	TrbF	-	-	WP_011970261.1
	COG3504	VirB9	QKN18446.1	WP_010967688.1	WP_011971094.1
	COG3704	VirB6	QKN18443.1	WP_013845459.1	WP_011971092.1
	COG3736	VirB8	QKN18445.1	WP_010967689.1	WP_011971093.1
	COG2948	VirB10	QKN18447.1	WP_010967687.1	WP_011971095.1
	COG3451	VirB4	-	-	-
	COG0630	VirB11	QKN18448.1	WP_010967686.1	-
	COG3505	VirD4	QKN18557.1	WP_010967483.1	WP_024325706.1
	COG3157	Hcp	-	-	-

## Data Availability

The data presented in this study are openly available in NCBI. The whole genome sequence of *Sinorhizobium meliloti* strain WSM1022 was deposited aunder BioProject PRJNA636618 with BioSample SAMN02597176, and GenBank/RefSeq assembly accession GCF_0013315775.1.

## Supplementary Materials

The following are available online at https://www.mdpi.com/article/10.3390/microorganisms9122428/s1, Figure S1: GenSkew GC skew plots. Figure S2: MAUVE multiple sequence alignment of the chromosome and large megaplasmids of the three rhizobial strains. Table S1: WSM1022 genome assembly metrics. Table S2: OrthoVenn2 results for all protein clusters. JSON format files containing the information required to reproduce Figure 1 are provided. They can be uploaded to http://cgview.ca/viewer (accessed on 10 October 2021) for a fully zoomable interactive plot.
