# Supplementary figures and images for: Comparative Genomics across Three Ensifer Species Using a New Complete Genome Sequence of the Medicago Symbiont Sinorhizobium (Ensifer) meliloti WSM1022
Source: Microorganisms. 2021 Nov 25;9(12):2428. doi: 10.3390/microorganisms9122428 (PMC8706082; doi:10.3390/microorganisms9122428)

A

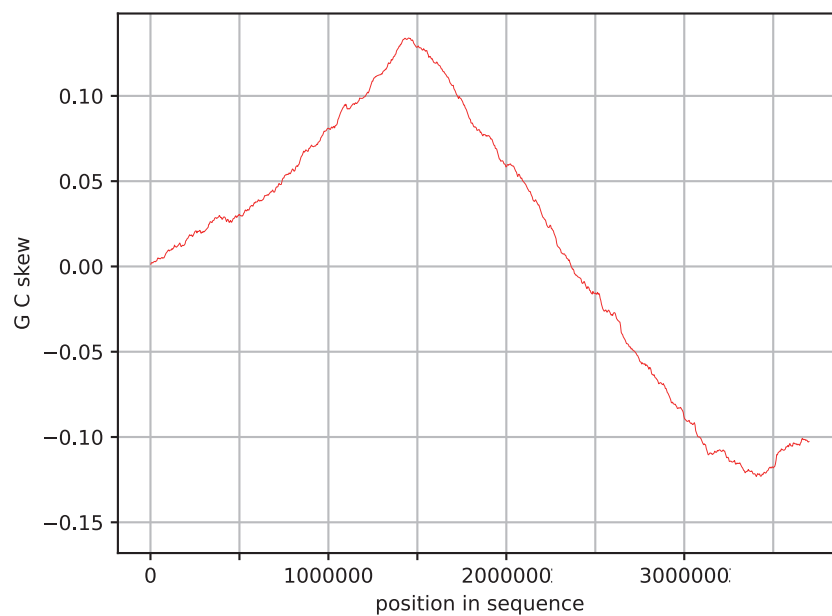

B

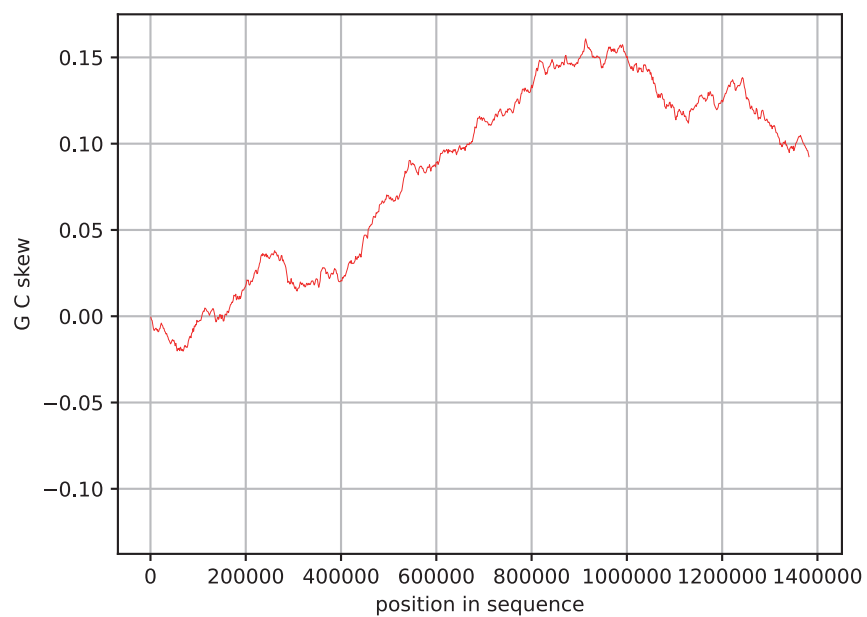

C

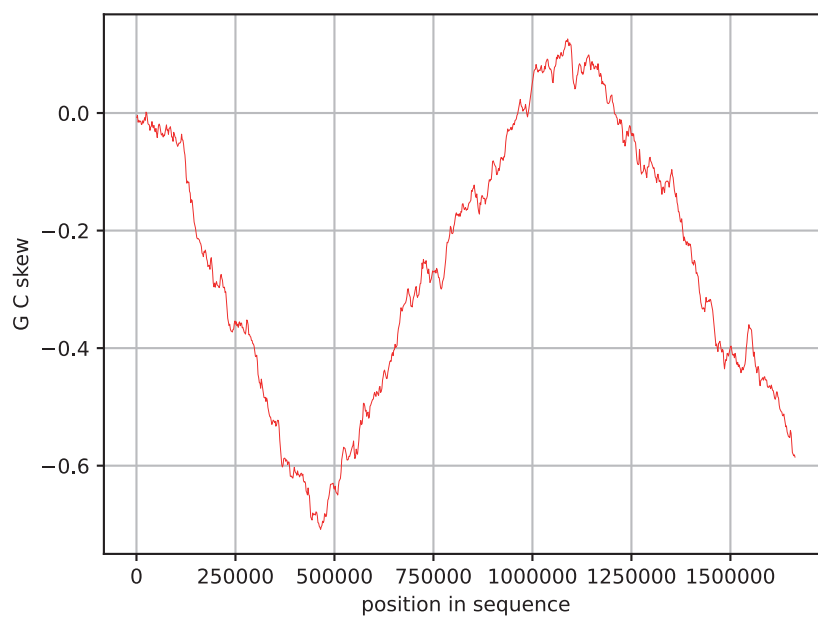

Supplement: Supplementary file 1 [file microorganisms-09-02428-s001.zip › suppl_files/FigureS1_NoLegend.pdf]

A

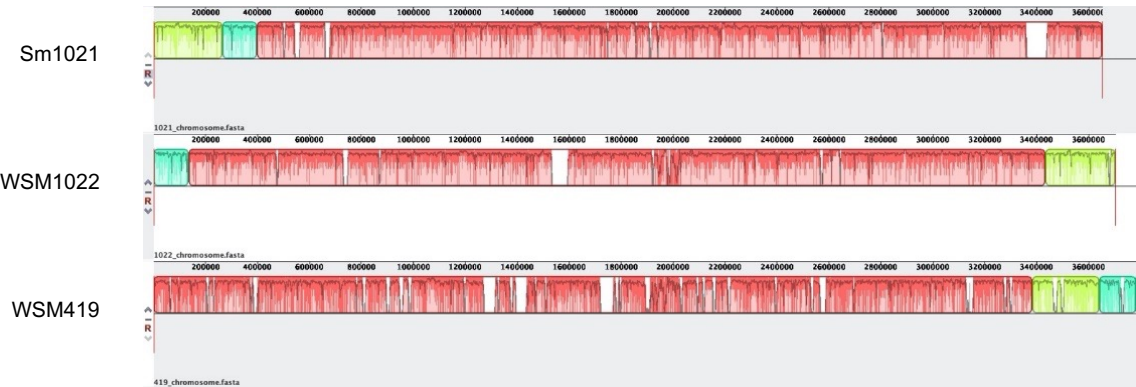

B

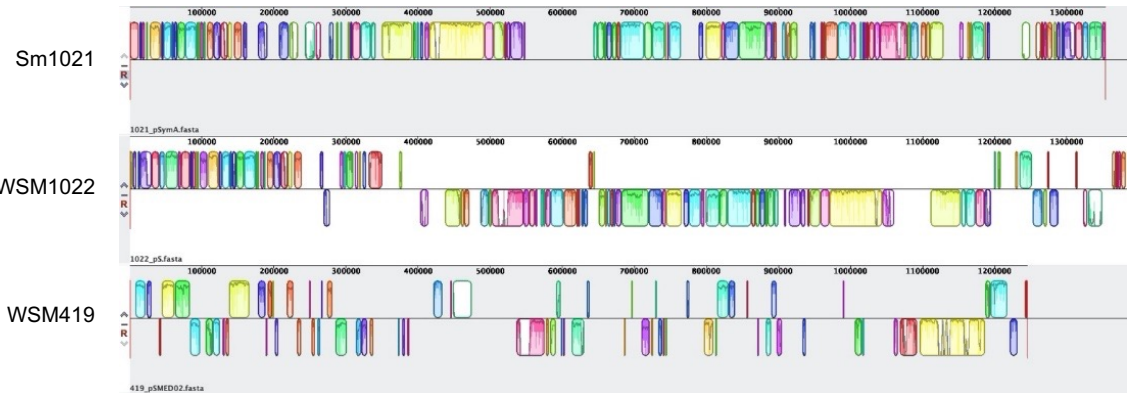

C

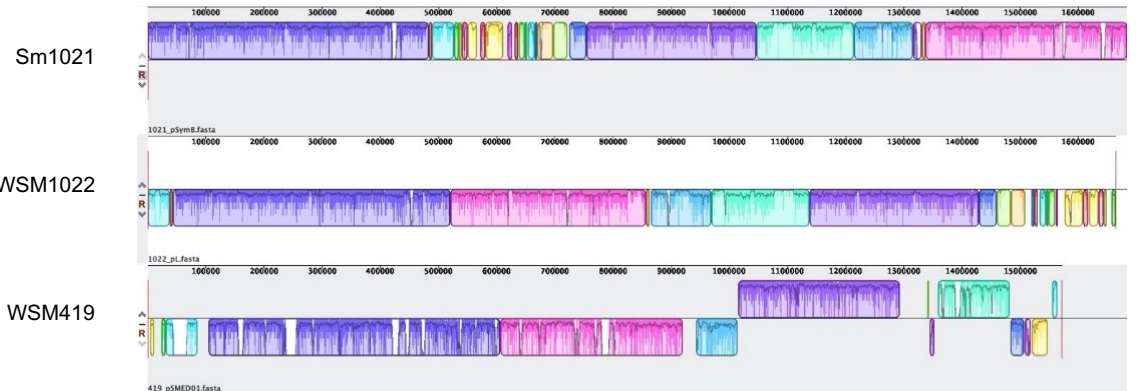

Supplement: Supplementary file 1 [file microorganisms-09-02428-s001.zip › suppl_files/FigureS2_NoLegend.pdf]

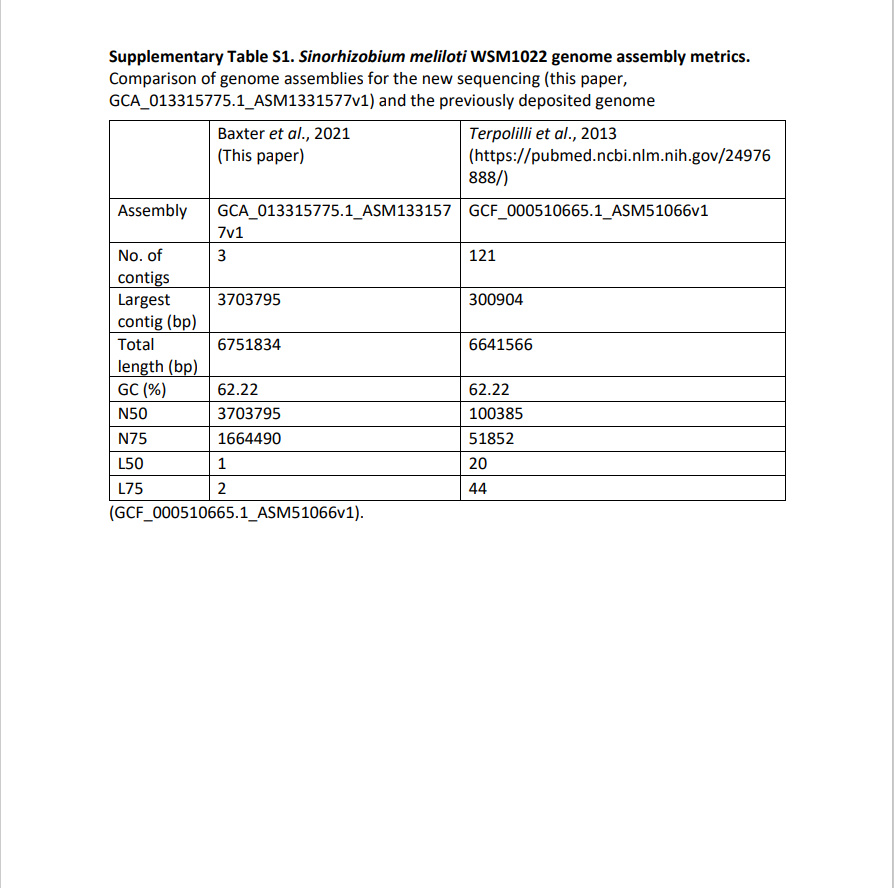

Supplement: Supplementary file 1 [file microorganisms-09-02428-s001.zip › suppl_files/Table S1.PNG]
